# Septal hypertrophy and cell cycle re-entry in AD

**DOI:** 10.18632/aging.101777

**Published:** 2019-01-22

**Authors:** Tracy Butler, Richard Bowen, Craig S. Atwood

**Affiliations:** 1 Center for Brain Health, Department of Psychiatry, New York University School of Medicine, New York, NY10016, USA; 2 Department of Psychiatry, Medical University of South Carolina, Charleston, SC 29425, USA; 3 Department of Medicine, University of Wisconsin-Madison School of Medicine and Public Health, Madison, WI 53705, USA

**Keywords:** Alzheimer’s Disease, magnetic resonance imaging, basal forebrain, luteinizing hormone, septal nuclei

Using MRI, we recently demonstrated enlargement of basal forebrain septal nuclei in healthy, cognitively-intact subjects destined to develop Alzheimer’s Disease (AD) in an average of approximately three years [1]. Others have shown enlargement/thickening of medial temporal lobe gray matter structures including the hippocampi in subjects with AD pathology but without cognitive impairment [2]. Septal nuclei and hippocampi are structurally and functionally closely connected via the fimbria/fornix and together are critical for memory performance. Enlargement of these structures in association with risk of AD is counterintuitive, as AD is a neurodegenerative disease associated with *loss* of gray matter volume.

What could be happening in the septal and hippocampal regions to cause enlargement prior to the development of cognitive decline? We suggest the following possibilities:

1. A form of cognitive/neuroanatomic reserve by which pre-existing larger gray matter structures are associated with better cognitive performance, which delays or prevents manifestation of dementia.

2. Neuroplastic compensation perhaps mediated by neurotrophins (NGF and BDNF) and related to the propensity of basal forebrain cholinergic neurons to express neurotrophin receptors throughout life and to enlarge via a growth factor-mediated process in response to hippocampal pathology and/or exogenous neurotrophic administration [3].

3. Amyloid deposition

4. Inflammation

5. Cell cycle dysregulation with failed replication, polyploidy and cellular hypertrophy [4].

Of these possibilities, cell cycle dysregulation is of particular interest because it represents a model of AD pathogenesis that has received limited attention, but which has important therapeutic implications. According to this model, mature neurons in regions such as basal forebrain and hippocampus attempt cell division but fail, resulting in excess DNA, cellular hypertrophy and ultimately, cell death [5]. Understanding the signal for neurons to enter this fatal attempt at replication is critical. While amyloid deposition, NGF and inflammation may play a role, there is strong evidence that Luteinizing Hormone (LH), a reproductive hormone which rises dramatically after menopause and during andropause, may be a primary driver for aberrant cell cycle entry. LH is elevated in the blood and CSF of AD patients above that of age-matched controls, and genetic or other interventions which block LH signaling prevent amyloid deposition, tau phosphorylation and neurodegeneration in multiple animal models of AD [4]. Moreover, LH is a known regulator of growth factors (NGF, BDNF, GDGF) required for oocyte maturation in the ovary. A phase II clinical trial of Leuprolide Acetate (Lupron), a GnRH analogue which decreases LH levels, benefitted cognition in a subgroup of women with AD who were taking an acetylcholinesterase inhibitor [6]. We have begun a new trial to replicate this result (https://clinicaltrials.gov/ct2/show/NCT03649724).

It will be necessary to more closely link MRI studies with pathology to know whether MRI-visible gray matter enlargement demonstrated by us and others in patients prior to the development of AD corresponds to cell body hypertrophy and polyploidy seen neuropathologically. This will be difficult in human patients but is amenable to animal study. Better understanding of the nature, time course and clinical associations of cellular and MRI-visible hypertrophy of gray matter structures such as septal nuclei and hippocampi can help explain why structural MRI changes in association with AD treatment are often in the wrong direction, i.e. showing greater gray matter volume loss and ventricular enlargement *with* treatment [7]. A validated structural MRI biomarker of cellular hypertrophy due to failed cell replication would be very useful in assessing the effectiveness of potential AD therapies, especially those such as Lupron premised on the cell cycle theory of AD.
